# Understanding the influence of 24-hour movement behaviours on the health and development of preschool children from low-income South African settings: the SUNRISE pilot study

**DOI:** 10.17159/2078-516X/2020/v32i1a8415

**Published:** 2020-01-01

**Authors:** C E Draper, S A Tomaz, C J Cook, S S Jugdav, C Ramsammy, S Besharati, A van Heerden, K Vilakazi, K Cockcroft, S J Howard, A D Okely

**Affiliations:** 1 South African MRC/Wits Developmental Pathways for Health Research Unit, Faculty of Health Sciences, University of the Witwatersrand, Johannesburg, South Africa; 2 Division of Exercise Science and Sports Medicine, UCT Research Centre for Health through Physical Activity, Lifestyle and Sport, Department of Human Biology, Faculty of Health Sciences, University of Cape Town, South Africa; 3 Faculty of Health Sciences and Sport, University of Stirling, UK; 4 Department of Psychology, University of the Witwatersrand, South Africa; 5 Human Sciences Research Council, South Africa; 6 Early Start, Faculty of Social Sciences, University of Wollongong, Australia

**Keywords:** early childhood development, physical activity, screen time, sleep

## Abstract

**Background:**

The International Study of Movement Behaviours in the Early Years, SUNRISE, was initiated to assess the extent to which young children meet movement behaviour guidelines (physical activity, sedentary behaviour, screen time, sleep).

**Objective:**

The South African SUNRISE pilot study assessed movement behaviours in preschool children from two low-income settings, and associations between these movement behaviours, adiposity, motor skills and executive function (EF).

**Methods:**

Preschool child/parent pairs (n = 89) were recruited from preschools in urban Soweto and rural Sweetwaters. Height and weight were measured to assess adiposity. Physical activity was assessed using accelerometers while sedentary behaviour, screen time and sleep were assessed via parent report. Fine and gross motor development were measured using the Ages and Stages Questionnaire-3, and EF was assessed using the Early Years Toolbox.

**Results:**

The proportion of children meeting the physical activity guideline was 84%, 66% met the sleep guideline, 48% met the screen time guideline, and 26% met all three guidelines. Rural children were more active, but spent more time on screens compared to urban children. Most children were on track for gross (96%) and fine motor (73%) development, and mean EF scores were in the expected range for all EF measures. EF was negatively associated with screen time, and gross motor skills were positively associated with physical activity.

**Conclusion:**

The South African SUNRISE study contributes to the growing literature on 24-hour movement behaviours in SA preschool children, and highlights that these behaviours require attention in this age group.

In 2018, the South African (SA) 24-hour movement guidelines for birth to five years were released.^[1]^ These guidelines recommend that preschool children should, in a day, spend an average of 180 minutes in total physical activity (TPA) which includes 60 minutes of ‘energetic play’ (moderate- to vigorous-intensity physical activity, MVPA), engage in less than one hour of screen time, and get 10–13 hours of sleep.^[1]^ These guidelines align with the World Health Organisation (WHO),^[2]^ Canada, and Australia, who all have 24-hour integrated guidelines for young children; and they align with the United Kingdom guidelines on physical activity in the early years. Development of these guidelines stems from the recognition that movement behaviours play a foundational role in the prevention and management of childhood obesity and noncommunicable diseases, as highlighted by the WHO Ending Childhood Obesity report, as well as the importance of healthy movement behaviours for optimal development in childhood.^[3]^

The development of global and national guidelines has highlighted the need for appropriate surveillance methods. This led to SUNRISE – an International Study of Movement Behaviours in the Early Years (https://sunrise-study.com). South Africa was one of the first countries to participate in the SUNRISE pilot study. The primary aim of the main SUNRISE study is to determine the proportion of children sampled in participating countries who meet the WHO Global Guidelines for physical activity, sedentary behaviour and sleep for children under five years of age. Secondary aims are to: (1) determine if proportions differ by sex, urban/rural location or between different levels of human and economic development; and (2) assess associations between movement behaviours and indicators of motor and cognitive development.

This paper on the South African SUNRISE pilot study presents descriptive findings on movement behaviours in preschool children from two low-income settings (rural and urban), and the associations between these movement behaviours, adiposity, motor skills and cognitive development. This paper also comments briefly on the feasibility and acceptability of the SUNRISE outcome measures.

## Methods

### Study settings and recruitment

Data were collected from two low-income settings in South Africa: urban Soweto, Johannesburg (Gauteng), and rural Sweetwaters (KwaZulu-Natal). Children and parents (or primary caregivers, n = 89 child/parent pairs) were recruited from four preschools in Soweto, and three preschools in Sweetwaters. Written informed consent was obtained from parents for all children and parents consented to complete the SUNRISE parent questionnaire. Ethical approval for this study was obtained from the Human Research Ethics Committee (Medical) at the University of the Witwatersrand (ref: M180490).

### Measures and procedures

All data collection with children took place at the preschools during the preschool day. Parent questionnaires were interviewer-administered and completed at a time convenient for parents, either at home or their child’s preschool. Data were collected by trained fieldworkers. In Sweetwaters, the questionnaire was administered in isiZulu and in Soweto it was administered in English, with explanation in local languages where necessary.

### Anthropometrics

The children’s height and weight were measured using a portable stadiometer (Leicester 214 Transportable Stadiometer; Seca, Germany) and a calibrated scale (Soehnle 7840 Mediscale Digital, Soehnle Industrial Solutions, Germany). All measurements were taken twice, and an average was used for analysis. Height and weight were used to calculate the Body Mass Index (BMI). BMI and associated z-scores were computed using the WHO AnthroPlus software (http://www.who.int/growthref/tools/en/). International Obesity Task Force cut-offs ^[4]^ were applied to BMI scores to classify children as thin, normal weight, overweight, and obese.

### Accelerometry

Physical activity was measured using hip-worn Actigraph GT3X+ accelerometers (Actigraph LLC, Pensacola, FL; USA). The device was set to start recording at midnight on the day of fitting and was collected four days later to ensure 72 continuous hours of wear. ActiLife v.6 (ActiLife software; Pensacola, FL; USA) was used to download in 15-second epochs, clean and score all accelerometry data. Visual inspection was conducted to determine if participants wore the device for a minimum of 24-hours. A predetermined time filter (5 am to 11.30 pm) was applied to all ‘valid’ days to avoid sleeping time being classified as non-wear or sedentary time. Only data recorded for each ‘valid’ day were considered for analyses. Non-wear time (including daytime naps, and when device was removed for bathing) was defined as 20 minutes or more of consecutive zeroes and was removed.^[5]^ Cut points used for light-intensity physical activity (LPA) and moderate- to vigorous-intensity physical activity (MVPA) were ≥200 counts.15s^−1^ and >420 counts.15s^−1^, respectively.^[6–7]^ Duration of time spent in LPA, MVPA and total physical activity (TPA) were determined.

### Motor skills

The Ages and Stages Questionnaire-3 (48 months, ASQ-3), a developmental screening assessment,^[8]^ was used to assess gross motor and fine motor skills. It has a categorical output for each motor skill component (child requires follow-up and further assessment/action, child is developing on schedule but may benefit from extra practice in some of the skills, child is developing on schedule).

### Executive function

Executive function (EF) is the marker of cognitive development used in SUNRISE and refers to the cognitive control processes that enable working with mental information (working memory), while resisting distractions and contrary impulses (inhibition) and flexibly (re-) directing attention as needed (shifting).^[9]^ EF in the early years has been found to predict lifelong achievement, health, wealth, quality of life, academic achievement and school readiness.^[9]^ The iPad-based Early Years Toolbox (EYT) was used to assess EF.^[10]^ This is available in five local South African languages (isiZulu, Sesotho, isiXhosa, Xitsonga and Afrikaans), takes ~20 minutes to complete per child, and includes assessments of working memory, inhibition and shifting. The translated versions have been used previously.^[11]^ An exploratory factor analysis (EFA)-derived factor score (EF composite score) was computed for these three EYT tasks so as to not constrain the number of planned analyses, and to more purely index EF (than any single EF measure in isolation). Inter-task correlations (coefficients from 0.18 to 0.43) were similar to those previously reported (where, Ref?), and EFA supported their combination.^[10]^

### SUNRISE parent questionnaire

The interviewer-administered SUNRISE parent questionnaire covered the following in relation to the child, for the previous week: PA (total PA, and moderate- to vigorous-intensity PA, MVPA), time spent outside, screen time and use of screens before bedtime, time spent restrained (strapped in and unable to move), time spent sitting, and sleep (typical hours slept, bedtime routine). Parents were also asked how they used screens with their child, how often they read to their child, and the highest education level in household.

### Statistical analysis

Data were analysed using SPSS Statistics for Windows (V 25.0). Continuous data were presented as mean ± SD if normally distributed or median (interquartile range) if not. To examine differences between boys and girls, and rural and urban, Mann-Whitney-U tests (for continuous variables) and Pearson’s Chi^2^ test (for categorical variables) were performed. To test for associations between variables, Spearman’s rank correlation coefficients were calculated. Kruskal-Wallis tests were conducted to examine differences in BMI-for-age z score (BAZ), gross motor skills, fine motor skills, and EF (EFA-derived factor) between children meeting one, two or three guidelines. Children were classified as meeting PA guidelines if they spent an average of 180 minutes per day in TPA, inclusive of 60 minutes per day of MVPA (objectively measured). They were classified as meeting the screen time guideline if they had less than one hour per day of parent-reported screen time, and were getting 10–13 hours of parent-reported sleep per 24-hour day.

## Results

The final sample used for analysis comprised 88 child/parent pairs (41 girls, 47%;47 boys, 53%; 39 urban Soweto, 44%; 49 rural Sweetwaters, 56%). Table 1 summarises children’s age and anthropometric characteristics. There were no significant differences between boys and girls for age or any anthropometric outcomes (all p > 0.05). The only significant difference between the urban and rural sub-samples was for height-for-age z score (HAZ) (p = 0.038). Using WHO cut-offs, 67% of the sample were in the normal range for BMI, 22.7% were identified as being at ‘possible risk of overweight’, and 5.7% and 4.5% were overweight and obese, respectively. Using International Obesity Task Force (IOTF) cut-offs, 72% of the sample were classified as normal weight, 11% as overweight, 7% as obese, and 10% as thin.

Table 2 summarises the accelerometry, motor skills and EF results. Boys and girls were similar for all PA variables (all p > 0.05). Rural children had significantly less sedentary time than urban children (p = 0.001), as well as being more physically active (LPA p < 0.001; MPA p < 0.001; vigorous-intensity physical activity (VPA) p = 0.008; MPVA p < 0.0005 and TPA p < 0.001). Boys and girls were similar for both gross and fine motor skill scores (both p > 0.05), but compared to rural children, urban children had significantly higher fine motor skill scores (p < 0.001). For gross motor skills, 96% of children scored in the highest category (‘developing on schedule’). For fine motor skills, 14% of children scored in the poorest category (‘requires follow-up and further assessment/action’), and 73% in the highest category. For all EF variables, boys and girls were similar (p > 0.05 for all variables). Rural children had significantly lower scores compared to urban children for working memory (p < 0.001), shifting (p < 0.001) and the EF composite score (p < 0.001).

**Fig. 1:**
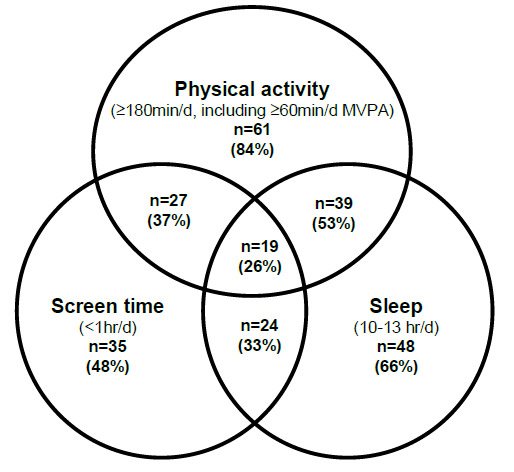
Venn diagram illustrating the proportion of children meeting 24-hour movement guideline components (valid n=73)

Figure 1 illustrates the number of children meeting the different components of the 24-hour movement guidelines. Amongst participants with valid data for all three components (83% of the sample, n = 73), most parents (89%) reported that, in the past week, their child had not been restrained for more than 60 minutes at a time, thus meeting this other component of the sedentary behaviour guideline. When looking at the guidelines separately for the full sample, 65 participants (84% of n = 77 valid) met the PA guideline, 40 (47% of n = 85 valid) met the screen time guideline, and 54 (65% of n = 83 valid) met the sleep guideline.

**Fig. 2:**
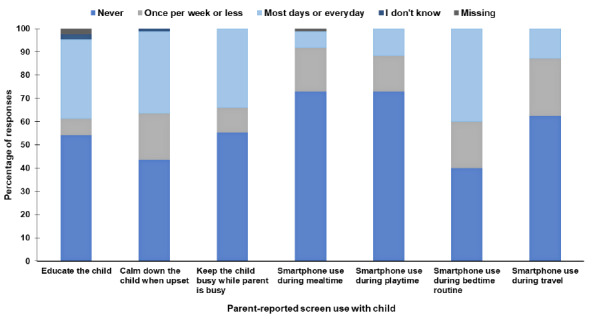
Frequency of parent-reported screen use with the child

Results of the parent questionnaire are presented in Table 3. There were no differences between boys and girls for any parent-reported behaviours. Parents in the rural setting reported more PA (p < 0.001) energetic play (p = 0.007), screen time (p = 0.002) and time spent sitting (p < 0.001). Parent-reported screen time practices with the child are shown in Figure 2. The percentage of parents who indicated that their child used a screen before bed was 82%, and 49% indicated that there was a screen in the room where the child sleeps. Approximately half of the parents reported that their child had consistent bedtimes (52% responded ‘yes’) and 70% of parents reported that their child has consistent wake up times. Only 10% of parents reported reading to their child every day, and in response to the question: How many days did you or other household members read to this child? 53 parents (60%) reported an average of 3.5±2.3 days.

Correlation results indicated associations between some variables. EF was negatively associated with BAZ (r_s_ = -0.31, p = 0.004), gross motor skills (r_s_ = −0.24, p = 0.036), and screen time (r_s_ = −0.31, p = 0.004) and positively associated with fine motor skills (r_s_ = 0.61, p < 0.001). BAZ was positively associated with TPA (r_s_ = 0.25, p = 0.03) and screen time (r_s_ = 0.22, p = 0.039). Gross motor skills were positively associated with both TPA (r_s_ = 0.30, p = 0.010) and MVPA (r_s_ = 0.28, p = 0.019). Fine motor skills were negatively associated with TPA (r_s_ = −0.30, p = 0.011). Sleep was not associated with any variables. As indicated in Table 4, the results of the Kruskal-Wallis test showed no significant differences. However, the mean ranks show that scores are in the expected direction: when two or three guidelines were met, children appeared to have lower BAZ, better gross motor skills and EF skills compared to children who only met one guideline.

Regarding the feasibility and acceptability of the SUNRISE outcome measures, most measures have been used successfully in previous South African studies with this age group, and performed well in this study. These included anthropometric measures, use of the hip-worn Actigraph GT3X+ accelerometers, and the EYT. This is not the first study to use the ASQ-3 in South Africa, but it confirmed that it is a feasible and acceptable measure of motor skills in low-income South African settings. Lastly, the SUNRISE parent questionnaire was feasible and acceptable in these settings, only if it was administered by a fieldworker. Parents found it difficult to report on their child’s PA while at preschool, which suggests that parent-reported PA levels should be interpreted with caution, and that the SUNRISE main study in South Africa should rely on objectively-measured PA for this age group.

## Discussion

The South African SUNRISE pilot study is the first in the country to include all three movement behaviours, to publish parent-reported screen time in preschool-aged children, and to examine associations between screen time, sleep and other early childhood development outcomes. This study confirms previous findings that overweight and obesity need to be addressed in this age group of South African children, taking into consideration the double burden of over- and undernutrition that has been noted in previous national and regional studies.^[12–14]^

In the South African SUNRISE sample, levels of TPA were lower than in previous studies in this age group from similar settings, although levels of MVPA are comparable, at least in terms of meeting the MVPA guideline.^[11,14–15]^ The difference in TPA is likely due to a higher cut point for LPA used for the SUNRISE sample, meaning that what had been classified as LPA in previous studies, is in this study more likely to be classified as sedentary behaviour. Although close to two-thirds of children in the SUNRISE sample met the sleep guideline, parent-reported sleep is likely to overestimate actual sleep time. A previous study in Soweto that measured sleep objectively in preschool children supports this likely overestimation, since it highlighted late bedtimes and that the majority of children were reliant on daytime naps to meet the guidelines.^[14]^ The SUNRISE pilot findings regarding the consistency of bedtimes (only 52%) and screen use before bed (82%), included as part of the bedtime routine (60%), add to the need to address sleep behaviour in this age group in low-income South African settings.

Less than half of the sample met the screen time guideline, which aligns with the global trend of high proportions of young children exceeding screen time guidelines, including those in low- and middle-income countries. While educating parents about South Africa’s guidelines,^[1]^ it is clear from these findings that parents in low-income South African settings need support on parenting strategies that do not involve screens, for example, when needing to calm a child down when upset, and keeping a child busy. Given that only 10% of parents reported reading to their child, it is possible that encouraging parents to do this, especially as part of their bedtime routine, could be beneficial for reducing screen time, improving early learning outcomes, and encouraging nurturing interactions between parents and children.^[1]^

The gross motor performance of these children aligns with previous research from low-income South African settings that found preschool children perform well in this domain.^[11,16]^ EF in the South African SUNRISE sample is also comparable to previous South African studies using the EYT in this age group, which found that children perform within and, in some cases exceed, the normal range for EF, and that urban children have better working memory and shifting than rural children.^[11]^ While possible reasons for better than expected EF have been hypothesised,^[11]^ further research is required to better understand young children’s EFs in low-income South African settings. Although there were no significant differences for BAZ, motor skills and EF for children meeting one, two or three guidelines, correlation results were revealing: children with lower screen time demonstrated better EF, and children who engaged in more PA demonstrated better gross motor skills. However, given the cross-sectional nature of this study, it is possible that these relationships are bidirectional and causality cannot be inferred. Associations between BAZ, EF, TPA and gross motor skills need further investigation using larger samples to better understand the effects of meeting specific combinations of the guidelines.

This is the first study to investigate the association between EF and fine motor skills in South African preschool children, and may indicate activities that develop fine motor skills, e.g. playing with blocks, puzzles, colouring in and drawing, are also beneficial for EF. This supports the message of South Africa’s guidelines^[1]^ that encourage these activities. The negative association between fine motor skills and TPA could be an indication that PA could be displacing activities for developing fine motor skills, or that children who do not have access to resources for fine motor activities choose active play as an alternative. However, this finding could also be explained by limited variance in TPA within the sample (very few children in the sample were engaging in less than 180 minutes of TPA per day). A larger sample with greater variability could more accurately determine the nature of this relationship.

The strengths of this study include the use of well-established measures for this age group in low-income South African settings, and the benefit of the collective expertise and experience of the SUNRISE global leadership group to inform on the study design. The main limitation is the small sample size and that the settings are not nationally representative. However, these sites benefited from the research capacity and existing relationships that helped to facilitate community engagement. When planning for the main South African SUNRISE study, engagement with the community will form a crucial component of its planning and execution. Furthermore, this pilot study provided useful insights into recruitment, data collection ‘trouble-shooting’, and other methodological considerations for the main SUNRISE study in South Africa. Although it will not be feasible to recruit a nationally representative sample for the main SUNRISE study, the sample size will be substantially larger (~1000 child/parent pairs) and more diverse.

## Conclusion

The South African SUNRISE pilot study contributes valuable initial findings to the growing literature on 24-hour movement behaviours in South African preschool children, and highlights that these behaviours require attention in this age group. This is particularly important considering the ubiquity of screens and the internet in many areas of South Africa. Understanding how movement behaviours are associated with key outcomes in early childhood is vital for setting this country’s children on their best trajectories for health and early learning.

## Figures and Tables

**Table 1:** Children’s age and anthropometric characteristics, by sex and setting

	Total (n = 88)	Boys (n = 47)	Girls (n = 41)	p value‡	Rural (n = 49)	Urban (n = 39)	p value§
**Age (y)**	4.5 ± 0.3	4.5 ± 0.4	4.5 ± 0.3	0.964	4.5 ± 0.3	4.5 ± 0.4	0.282
**Height (cm)**	103.5 ± 4.6	103.6 ± 5.1	103.4 ± 4.1	0.802	102.9 ± 4.8	104.3 ± 4.3	0.183
**Weight (kg)**	17.6 ± 3.0 18.6 (15.7–18.9)	17.5 ± 2.6 17.2 (15.8–18.6)	17.6 ± 3.3 16.7 (15.5–18.7)	0.544	17.6 ± 3.1 16.8 (15.6–18.6)	17.5 ± 2.8 17.3 (15.7–18.7)	0.857
**BMI (kg.m** **^−2^** **)**	16.3 ± 2.2 15.9 (15.2–17.1)	16.3 ± 1.9 16.2 (15.2–17.1)	16.4 ± 2.5 15.7 (15.3–17.1)	0.910	16.6 ± 2.2 16.3 (15.5–17.2)	16.0 ± 2.1 15.4 (14.7–16.9)	0.063
**HAZ**	−0.65 ± 0.90	−0.69 ± 0.97	−0.61 ± 0.82	0.649	−0.83 ± 1.10	−0.43 ± 0.77	0.038*
**WAZ**	0.02 ± 1.06 −0.10 (−0.68–0.53)	−0.01 ± 1.00 −0.10 (−0.65–0.53)	0.07 ± 1.15 −0.10 (−0.69–0.55)	0.943	0.02 ± 1.06 −0.01 (−0.68–0.47)	0.02 ± 1.08 −0.09 (−0.68–0.54)	0.857
**BAZ**	0.65 ± 1.30 0.39 (−0.07–1.27)	0.66 ± 1.22 −0.10 (−0.69–0.55)	0.64 ± 1.39 0.33 (0.05–1.16)	0.757	0.82 ± 1.30 0.67 (0.14–1.34)	0.44 ± 1.27 0.14 (−0.40–1.07)	0.057

Data are presented as mean ± SD for normally distributed data; not normally distributed data includes median (25^th^–75^th^ percentile) for the total sample.

* indicates significance at p < 0.05;

‡ p value for comparison by sex;

§ p value for comparison by setting.

BMI, body mass index; HAZ, height-for-age z score; WAZ, weight-for-age z score; BAZ, BMI-for-age z score.

**Table 2:** Accelerometry, motor skill and executive function results, by sex and setting

	Total (n = 77)	Boys (n = 40)	Girls (n = 37)	p value‡	Rural (n = 43)	Urban (n = 34)	p value§
**SB (min/d)**	748 ± 83	752 ± 83	745 ± 84	0.743	723 ± 75	782 ± 82	0.001*
**LPA (min/d)**	127 ± 28	124 ± 29	129 ± 28	0.426	140 ± 24	110 ± 25	<0.001*
**MPA (min/d)**	98 ± 30	101 ± 30	95 ± 31	0.434	110 ± 28	83 ± 26	<0.001*
**VPA (min/d)**	30 ± 15 28 (20–39)	32 ± 16 30 (21–42)	27 ± 12 27 (17–34)	0.114	34 ± 16 30 (24–43)	25 ± 11 23 (17–32)	0.008*
**MVPA (min/d)**	128 ± 43	133 ± 44	122 ± 41	0.249	144 ± 42	108 ± 35	<0.001*
**TPA (min/d)**	254 ± 67	257 ± 70	251 ± 65	0.692	283 ± 61	218 ± 56	<0.001*

	**Total (n = 88)**	**Boys (n = 47)**	**Girls (n = 41)**	**p value** ‡	**Rural (n = 49)**	**Urban (n = 39)**	**p value** §

**Gross motor skills** †	56 ± 10 60 (60–60)	56 ± 11 60 (60–60)	56 ± 10 60 (60–60)	0.342	57 ± 12 60 (60–60)	55 ± 8 60 (53–60)	<0.001*
**Fine motor skills** †	44 ± 16 50 (30–55)	42 ± 16 45 (30–55)	46 ± 16 50 (36–55)	0.166	36 ± 17 40 (20–50)	52 ± 9 55 (50–55)	<0.001*
**Working memory** †	3.4 ± 2.6 1.3 (0.3–2.0)	3.4 ± 2.8 1.3 (0.0–2.3)	3.5 ± 2.3 1.3 (0.3–2.0)	0.972	2.1 ± 2.4 0.3 (0.0–1.7)	5.0 ± 1.7 2.0 (1.3–2.3)	<0.001*
**Inhibition**	0.57 ± 0.19	0.57 ± 0.18	0.57 ± 0.20	0.965	0.54 ± 0.15	0.61 ± 0.23	0.089
**Shifting**	2.20 ± 3.47 0.00 (0.00–3.00)	2.19 ± 3.54 0.00 (0.00–3.00)	2.22 ± 3.43 0.00 (0.00–3.00)	0.771	0.37 ± 0.93 0.00 (0.00–0.00)	4.51 ± 4.08 3.00 (0.00–9.00)	<0.001*
**EF composite score** †	0.007 ± 1.005 −0.036 (−0.835–0.598)	−0.007 ± 1.096 −0.125 (−0.959–0.605)	0.023 ± 0.897 0.081 (−0.723–0.585)	0.636	−0.532 ± 0.683 −0.708 (−1.131–0.017)	0.657 ± 0.949 0.441 (0.046–1.556)	<0.001*

† n=86; 1 boy & 1 girl refused to complete tests; both from rural setting; data are presented as mean ± SD for normally distributed data;

# not normally distributed data includes median (25^th^–75^th^ percentile);

* indicates significance at p <0.001;

‡ p value for comparison by sex;

§ p value for comparison by setting.

SB, sedentary behaviour; LPA, light-intensity physical activity; MPA, moderate-intensity physical activity; VPA, vigorous-intensity physical activity; MVPA, moderate- to vigorous-intensity physical activity; TPA, total physical activity; EF, executive function; gross and fine motor skills are reported as Ages and Stages Questionnaire-3 scores; working memory, inhibition and shifting are reported as scores on the Early Years Toolbox tasks.

**Table 3:** Parent questionnaire results (continuous variables), by sex and setting

	Total (n = 85)	Boys (n = 45)	Girls (n = 40)	p value‡	Rural (n = 48)	Urban (n = 37)	p value§
**Parent/caregiver age (y)** #	35.2 ± 12.5 32.0 (27.0–41.0)	33.2 ± 8.8 37.0 (31.0–48.5)	37.4 ± 15.5 37.0 (29.5–47.3)	0.555	33.8 ± 12.5 30.0 (25.0–39.5)	36.9 ± 12.4 34.0 (28.0–44.5)	0.100
**TPA (h/d)** #	5.16 ± 2.34 5.0 (3.0–8.0)	4.95 ± 2.24 5.0 (3.0–6.0)	5.39 ± 2.46 5.5 (4.0–8.0)	0.365	6.31 ± 1.79 6.0 (5.0–8.0)	3.66 ± 2.13 4.0 (2.0–5.0)	<0.001*
**Energetic play (h/d)** #	2.84 ± 2.07 2.0 (1.0–4.0)	2.87 ± 1.94 3.0 (1.0–4.5)	2.80 ± 2.22 2.0 (1.0–4.0)	0.606	3.37 ± 2.14 3.0 (2.0–5.0)	2.16 ± 1.77 2.0 (1.0–3.5)	0.007**
**Screen time (h/d)** #	2.10 ± 1.73 1.5 (1.0–3.0)	2.32 ± 2.02 1.5 (1.0–3.0)	1.86 ± 1.32 1.8 (0.9–3.0)	0.568	2.69 ± 1.95 2.75 (1.0–3.8)	1.34 ± 0.97 1.0 (0.6–1.8)	0.002**
**Sleep (h/d)** †	10.29 ± 1.70	10.09 ± 1.91	10.52 ± 1.41	0.253	10.08 ± 1.73	10.55 ± 1.65	0.216
**Time spent sitting (h/d)** #	2.10 ± 2.34 1.0 (0.5–3.0)	1.97 ± 2.24 1.0 (0.4–3.0)	2.25 ± 2.47 1.0 (0.5–3.8)	0.497	3.00 ± 2.62 2.25 (0.5–5.0)	0.94 ± 1.17 0.75 (0.33–1.0)	<0.001*
**Time spent sitting in a vehicle (weekdays,h/d)** #	0.55 ± 1.41 0.0 (0.0–0.5)	0.59 ± 1.58 0.0 (0.0–0.5)	0.51 ± 1.20 0.0 (0.0–0.4)	0.256	0.70 ± 1.78 0.0 (0.0–0.5)	0.36 ± 0.64 0.0 (0.0–0.5)	0.666
**Time spent sitting in a vehicle (weekends,h/d)** #	0.34 ± 0.68 0.0 (0.0–0.42)	0.31 ± 0.61 0.0 (0.0–0.3)	0.38 ± 0.76 0.0 (0.0–0.9)	0.967	0.39 ± 0.76 0.0 (0.0–0.63)	0.28 ± 0.58 0.0 (0.0–0.33)	0.663

† n=83; 2 parents responses excluded; both parents of girls, from the rural setting; all data are presented as mean ± SD for normally distributed data;

# not normally distributed data includes median (25^th^–75^th^ percentile);

* indicates significance at p < 0.001;

** indicates significance at p < 0.05;

‡ p value for comparison by sex;

§ p value for comparison by setting.

TPA, total physical activity.

**Table 4:** Mean ranks of children meeting 1, 2, or 3 guidelines, for BAZ, gross motor skills, fine motor skills and executive function

Guidelines met	1 (n = 21) Mean rank	2 (n = 33) Mean rank	3 (n = 19) Mean rank	χ^2^	p value
**BAZ**	37.19	37.77	35.45	0.15	0.929
**Gross motor skills**	35.24	33.63	33.50	0.21	0.895
**Fine motor skills**	33.13	39.36	30.92	2.43	0.297
**EF composite score**	32.19	37.30	40.06	1.46	0.482

BAZ, BMI-for-age z score; EF, executive function
